# Mitochondrial DNA Variants of Respiratory Complex I that Uniquely Characterize Haplogroup T2 Are Associated with Increased Risk of Age-Related Macular Degeneration

**DOI:** 10.1371/journal.pone.0005508

**Published:** 2009-05-12

**Authors:** John Paul SanGiovanni, Dan E. Arking, Sudha K. Iyengar, Michael Elashoff, Traci E. Clemons, George F. Reed, Alice K. Henning, Theru A. Sivakumaran, Xuming Xu, Andrew DeWan, Elvira Agrón, Elena Rochtchina, Carolyn M. Sue, Jie Jin Wang, Paul Mitchell, Josephine Hoh, Peter J. Francis, Michael L. Klein, Emily Y. Chew, Aravinda Chakravarti

**Affiliations:** 1 National Eye Institute (NEI)/National Institutes of Health (NIH), Bethesda, Maryland, United States of America; 2 McKusick-Nathans Institute of Genetic Medicine, Johns Hopkins University, Baltimore, Maryland, United States of America; 3 Department of Epidemiology and Biostatistics, Case Western Reserve University, Cleveland, Ohio, United States of America; 4 The EMMES Corp., Rockville, Maryland, United States of America; 5 Department of Epidemiology and Public Health, Yale University School of Medicine, New Haven, Connecticut, United States of America; 6 Centre for Vision Research, Department of Ophthalmology, Westmead Millennium Institute, University of Sydney, Sydney, Australia; 7 Macular Degeneration Center, Casey Eye Institute, Oregon Health & Science University, Portland, Oregon, United States of America; 8 CardioDx, Palo Alto, California, United States of America; 9 University of Sydney Kolling Institute for Medical Research, Sydney, Australia; Louisiana State University, United States of America

## Abstract

**Background:**

Age-related macular degeneration (AMD), a chronic neurodegenerative and neovascular retinal disease, is the leading cause of blindness in elderly people of western European origin. While structural and functional alterations in mitochondria (mt) and their metabolites have been implicated in the pathogenesis of chronic neurodegenerative and vascular diseases, the relationship of inherited variants in the mitochondrial genome and mt haplogroup *subtypes* with advanced AMD has not been reported in large prospective cohorts.

**Methodology/Prinicipal Findings:**

We examined the relationship of inherited mtDNA variants with advanced AMD in 1168 people using a three-stage design on samples from 12-year and 10-year prospective studies on the natural history of age-related eye disease. In Stage I we resequenced the entire genome in 99 elderly AMD-free controls and 215 people with advanced AMD from the 12-year study. A consistent association with AMD in 14 of 17 SNPs characterizing the mtDNA T haplogroup emerged. Further analysis revealed these associations were driven entirely by the T2 haplogroup, and characterized by two variants in Complex I genes (A11812G of *MT-ND*4 and A14233G of *MT-ND6*). We genotyped T haplogroups in an independent sample of 490 cases and 61 controls from the same study (Stage II) and in 56 cases and 246 controls from the 10-year study (Stage III). People in the T2 haplogroup were approximately 2.5 times more likely to have advanced AMD than their peers (odds ratio [OR] = 2.54, 95%CI 1.36–4.80, *P*≤0.004) after considering the totality of evidence. Findings persisted after considering the impact of AMD-associated variants A69S and Y402H (OR = 5.19, 95%CI 1.19–22.69, *P*≤0.029).

**Conclusion:**

Loci defining the mtDNA T2 haplogroup and Complex I are reasonable targets for novel functional analyses and therapeutic research in AMD.

## Introduction

Age-related macular degeneration (AMD) is the leading cause of blindness in elderly people of western European ancestry [1], [2]. Evidence implicating the role of mitochondria (mt) in AMD pathogenesis has emerged over the past 2 decades [3] and the molecular genetics of this concept are now being investigated. A coding variant for respiratory Complex I defining the mitochondrial T haplogroup (4917G) was associated with an increased likelihood of having AMD in a U.S.-based study of 280 people with AMD and 280 age-matched controls [4]. While analyses from an Australian population-based cohort did not suggest association of any major European haplotype with advanced AMD, low accrual of endpoints, may have constrained inference [5]–[7]. Crosstalk between mitochondrial and nuclear genomes is a germane issue as independent teams report that the product of the AMD-associated LOC387715/ARMS2 of the nuclear genome localizes to mitochondria [8], [9]. While structural and functional alterations in mitochondria and their metabolites have been implicated in the pathogenesis of chronic neurodegenerative and vascular diseases [10], [11], the relationship of inherited variants in the mitochondrial genome and mt haplogroup *subtypes* with advanced AMD has not been reported in large prospective cohorts. We applied a rapid high-throughput method for this purpose on the basis of six lines of evidence: 1) cells most affected in AMD (retinal pigment epithelium (RPE) and photoreceptors) are densely packed with mitochondria; 2) ultrastructure of RPE and photoreceptor mitochondria is altered in people with AMD [12]; 3) mitochondrial metabolism yields the primary source of reactive oxygen and nitrogen species (RONS) in RPE and photorector cells [13]; 4) RONS target RPE and photoreceptor mitochondrial and nuclear DNA, chromatin, and lipids [13] and are capable of inducing somatic mutations that accumulate over time [11], [13]; 5) the retinal apoptosome is dominated by the mitochondrial pathway [13], [14]; and 6) our 3640-person multi-center clinical trial demonstrated efficacy of a number of antioxidant vitamins in reducing the likelihood of progression to sight-threatening advanced AMD [15]. We now report a 2.5-fold increased likelihood of having advanced AMD among people carrying variants in mt DNA T2 haplogroup-associated loci. The respective T2 variants are 11812G and 14233G of NADH ubiquinone oxidoreductase genes *MT-ND4* (mitochondrially encoded NADH dehydrogenase subunit 4) and *MT-ND6* (subunit 6).

## Results

**Figure 1:**
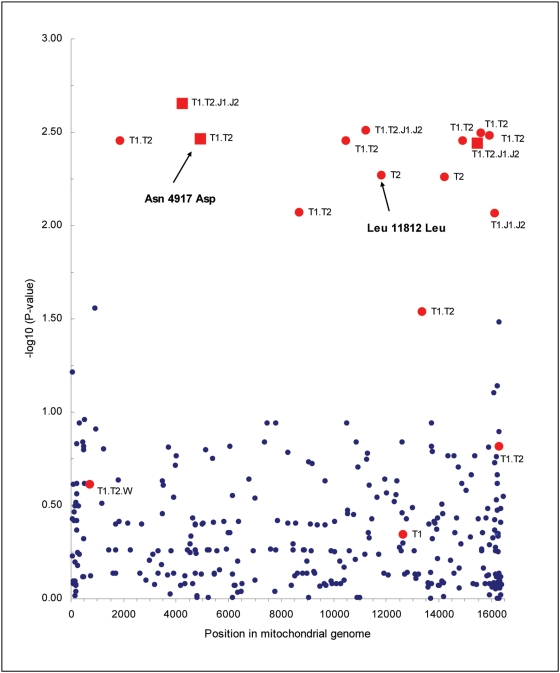
Distribution of age- and sex-adjusted P-values for all 315 mitochondrial DNA variants with minor allele frequencies >1% from the AREDS A cohort. *P*-values were obtained from logistic regression analyses modeling the likelihood of having increased frequency of the less commonly observed allele in people with advanced AMD. The magnitude of −log_10_ P-values was highest for SNPs partially or completely characterizing the T haplotype (see red symbols). Fourteen of the 17 variants in this category yielded the strongest associations in the entire sample. Among these T-haplotype-associated variants those represented by a square symbol lead to changes in protein structure. Details of these variants exist in Table 1. SNPs selected for analysis in our replication samples are indicated with arrows.

In Stage I, we applied a resequencing microarray to examine the complete sequence of the mitochondrial genome in a sample of 215 cases and 99 controls from The Age-Related Eye Disease Study (AREDS). The term ‘AREDS A’ is used to designate this cohort. We identified 998 unique variants and analyzed all 315 SNPs that were both called reliably at ≥90% of samples and present in at least 1% of cases or controls. Seventeen SNPs yielded associations with advanced AMD at *P*≤0.05 (**Table 1**). We were intrigued to observe that all 12 variants with *P*-values≤0.005 partially defined the mtDNA haplogroup T (**Figure 1**); nine of these SNPs uniquely characterized this haplogroup. A T haplotype-AMD relationship has been reported previously [4]. The T haplotype can be subdivided into T1 and T2 groups, with T1 defined by presence of the 4917G and 11812A variants and T2 defined by presence of 4917G and 11812G.

**Table 1:** Associations between mitochondrial variants and advanced AMD.

Variant locus	Gene	mt Function	Minor Variant Frequency (%)		OR (95%CI)	*P*-Value	Haplogroup
			No AMD	Adv. AMD			
73	Non-Coding	D-loop	48 (48)	77 (36)	0.58 (0.35–0.95)	0.030	H, HV, V
930	MT-RNR1	12S rRNA	1 (1)	19 (9)	12.52 (1.60–97.74)	0.016	–
1888	MT-RNR2	16S rRNA	3 (3)	33 (15)	7.92 (2.27–27.65)	0.001	T
4216	MT-ND1	Complex I	11 (11)	58 (27)	3.19 (1.56–6.53)	0.002	T, J
4917	MT-ND2	Complex I	4 (4)	35 (16)	6.15 (2.04–18.52)	0.001	T
8697	MT-ATP5	Complex V	5 (5)	34 (16)	4.84 (1.74–13.40)	0.003	T
10463	MT-TR	tRNA-Arg	3 (3)	33 (15)	7.92 (2.27–27.65)	0.001	T
11251	MT-ND4	Complex I	11 (11)	57 (26)	3.10 (1.51–6.35)	0.002	T, J
11812	MT-ND4	Complex I	2 (2)	29 (14)	10.45 (2.35–46.82)	0.002	T2
14233	MT-ND6	Complex I	2 (2)	29 (14)	10.35 (2.32–46.10)	0.002	T2
14905	MT-CYB	Complex III	3 (3)	33 (15)	7.92 (2.27–27.65)	0.001	T
15452	MT-CYB	Complex III	11 (12)	57 (27)	3.12 (1.51–6.40)	0.002	T, J
15607	MT-CYB	Complex III	3 (3)	34 (16)	8.09 (2.33–28.14)	0.001	T
15928	MT-TT	tRNA-Thr	3 (3)	34 (16)	8.02 (2.30–27.87)	0.001	T
16093	Non-Coding	D-loop	6 (6)	4 (2)	0.27 (0.07–1.00)	0.050	–
16304	Non-Coding	D-loop	3 (3)	22 (10)	3.95 (1.11–14.05)	0.034	H5
16126	Non-Coding	D-loop	13 (13)	58 (27)	2.51 (1.28–4.91)	0.007	T1, J

Odds ratios (ORs) are ratios of the odds of having the less commonly observed allele among people with advanced (Adv.) age-related macular degeneration (AMD) to the odds of having this variant within their AMD-free peers. Values higher than 1.0 suggest an increased likelihood of having advanced AMD associated with presence of the less commonly observed allele. Advanced AMD was classified as neovascular AMD or geographic atrophy of the macula (n = 215); No AMD (n = 99), these people were essentially free of age-related macular abnormalities with a drusen area of less than 5 small drusen (<63 um diameter) in both eyes; Models are age-, sex-, and smoking-adjusted. Age at the final fundus photograph used for phenotyping was modeled as a continuous variable; Complex I, NADH ubiquinone oxidoreductase;Complex III, ubiquinol cytochrome c oxidoreductase; Complex V, F1F0ATPase/ATP synthase; rRNA, ribosomal RNA; tRNA, transfer RNA. P-values are 2-sided and represent the minimum false positive rate when testing the hypothesis that people with AMD have different distributions of the less commonly observed allele than their AMD-free peers. Based on these results we examined distributions of SNPs A4917G and A11812G in an independent sample (see **Table 2**).

**Table 2** contains haplogroup frequencies and odds ratios (ORs) from analyses of T haplogroup-AMD relationships. Demographic characteristics of the original and replicate samples are given in **Table S1**. We applied multivariable logistic regression models to examine the likelihood that T1 and T2-associated loci occurred more frequently in people with advanced AMD than in AMD-free controls after simultaneously considering the impact of age-, sex-, and smoking history. People in the AREDS A cohort carrying the T1-defining variants (4917G/11812A) were not more likely than their peers to have advanced AMD (OR = 1.43, 95%CI 0.26–7.81, 2-sided *P*≤0.68). However, there was a 10.5-fold increased likelihood having advanced AMD if they carried the T2 variants (4917G/11812G, OR = 10.46, 95%CI 2.35–46.63, 2-sided *P*≤0.002).

**Table 2:** Measures of association for advanced AMD with variants characterizing T haplogroups.

Haplogroup	Sample Origin	Sample Size	Haplogroup	Frequency (%)	Multivariable OR (95% CI)	*P*-value*
		No AMD/Adv. AMD	No AMD	Adv. AMD		
**T1 (4917G/11812A)**						
	AREDS A	99/215	2 (2.02)	5 (2.33)	1.43 (0.26–7.81)	0.682
	AREDS B	60/480	4 (6.67)	15 (3.13)	0.64 (0.17–2.52)	0.265*
	AREDS A+B	159/695	6 (3.77)	20 (2.88)	0.97 (0.37–2.52)	0.946
	BMES	243/56	3 (1.23)	2 (3.57)	4.40 (0.69–27.96)	0.058*
	AREDS B+BMES	303/536	7 (2.31)	17 (3.17)	1.17 (0.33–4.26)	0.401*
	AREDS+BMES Combined Data	402/751	9 (2.24)	23 (3.06)	1.31 (0.50–3.39)	0.582
	AREDS+BMES Meta-analysis	402/751	9 (2.24)	23 (3.06)	0.96 (0.32–2.85)	0.943
**T2 (4917G/11812G)**						
	AREDS A	99/215	2 (2.02)	31 (14.42)	10.46 (2.35–46.63)	0.002
	AREDS B	61/490	1 (1.64)	39 (7.96)	6.12 (0.73–51.22)	0.048*
	AREDS A+B	160/705	3 (1.88)	68 (9.65)	5.26 (1.62–17.09)	0.006
	BMES	246/56	19 (7.72)	6 (10.71)	1.35 (0.50–3.64)	0.277*
	AREDS B+BMES	307/546	20 (6.51)	45 (8.24)	1.87 (0.83–4.02)	0.064*
	AREDS+BMES Combined Data	406/761	22 (5.42)	74 (9.72)	2.54 (1.36–4.80)	0.004
	AREDS+BMES Meta-analysis	406/761	22 (5.42)	74 (9.72)	3.40 (1.02–11.28)	0.046

Odds ratios (ORs) comparing the proportion of people with variants characterizing the T1 or T2 haplogroups and advanced (Adv.) AMD relative to their AMD-free peers. The mtDNA T1 haplogroup is defined by the presence of the 4917G variant and the presence of the 11812A variant. The T2 haplogroup is defined by the presence of 4917G and 11812G. The multivariable models are adjusted for age, sex, and smoking history (ever vs. never). Models for the AREDS+BMES cohorts contain a term for sample origin (study).

* P-values are 2-sided, unless marked with an asterisk; 1-sided P-values (for replication analyses) are for tests on the hypothesis that people with AMD are more likely to carry haplogroup-defining variants. All samples are composed of participants self-identified as being of non-Hispanic white origin. Demographic characteristics of the samples exist in **Table S1**. AREDS samples A and B are independent. ORs from the meta-analysis are from random effects models. Respective values for T1 and T2 from fixed-effects models are 0.85 (95%CI 0.37–1.96, 2-sided P<0.705) and 2.72 (95%CI 1.29–5.74, 2-sided P<0.009). AREDS = Age-Related Eye Disease Study. BMES = Blue Mountains Eye Study.

In Stage II we validated our findings by genotyping A4917G and A11812G in an independent AREDS sample of 490 cases and 61 controls. The term ‘AREDS B’ is used to designate this cohort. We used exact methods to confirm that allele frequencies for A4917G and A11812G were not significantly different across AREDS A and AREDS B. T1-associated SNPs were balanced across cases and controls in both samples. T2 distributions show that the advanced AMD group contained a higher proportion of people with 11812G and 4917G in each sample: 4917G/11812G existed in 2.02% of 99 controls vs. 14.42% of 215 cases in AREDS A and in 1.64% of 61 controls vs. 7.96% of 490 cases in AREDS B. Change in magnitude and precision of ORs was negligible after concurrent adjustment for age, sex, and smoking history. As in the AREDS A cohort, people in the T1 haplogroup were equally likely to have advanced AMD as their peers. The OR for advanced AMD in the sex-, age-, and smoking-adjusted model for T1 in AREDS B was 0.64 (95%CI 0.17–2.52, 1-sided *P*≤0.27). Results for the T2 association were also concordant: OR for advanced AMD in the sex-, age-, and smoking-adjusted model for T2 in AREDS B was 6.12 (95%CI 0.73–51.22, 1-sided *P*≤0.05). To obtain a more accurate assessment of AMD risk associated with the T2 haplogroup, we pooled samples from both AREDS A and AREDS B. As ORs were not different (*P* = 0.98 for T1 and 0.18 for T2, see Methods for details) and the study design was identical for AREDS A and AREDS B, we estimated associations in the entire sample. Results remained unchanged and precision was enhanced; for T2 OR = 5.26 (95%CI 1.62–17.09, 2-sided *P*≤0.006).

In Stage III we genotyped A4917G and A11812G in Blue Mountains Eye Study (BMES) participants with advanced AMD (n = 56) or who were ≥74 years-of-age and AMD-free (n = 246). T1 frequency was 1.23% (95%CI 0.45%–3.6%) in the AMD-free group and 3.57% (95%CI 1.10%–12.11%) in the advanced AMD group. Sex-, age-, and smoking-adjusted multivariable models yielded a non-significant increased odds of having advanced AMD among people carrying the T1-defining variants (OR = 4.40, 95%CI 0.69–27.96, 1-sided *P*≤0.058). T2 frequency was 7.72% (95%CI 5.0%–11.6%) for the AMD-free group and 10.71% (95%CI 5.1%–21.5%) for the advanced AMD group. T2-advanced AMD measures of association did not attain statistical significance within the BMES cohort (OR = 1.35, 95%CI 0.50–3.64, 1-sided *P*≤0.277). Combining our independent replication samples (AREDS B and BMES) yielded an OR = 1.87 (95%CI 0.83–4.02, 1-sided *P*≤0.064).

T2 haplogroup frequency for AREDS (8.21%, 95%CI 6.57%–10.23%) and BMES (8.28%, 95%CI 5.68%–11.94%) was similar and within limits reported from U.S. cohorts of European ancestry [16]. Table 2 indicates T2 frequency among people with AMD was also similar between studies. Applying the meta-analytic method of DerSimonian & Laird [17] yielded a combined OR of 3.40 (95%CI 1.02–11.28, 2-sided *P*≤0.046). We also combined raw data from AREDS A, AREDS B, and BMES in our final multivariable logistic regression models with a term for sample source (AREDS vs BMES), as differences in ORs for T2 between AREDS and BMES cohorts were not statistically different (*P* = 0.08). T2-AMD results persisted and were of the same magnitude of those from the DerSimonian & Laird estimates (OR = 2.54, 95%CI 1.36–4.80, 2-sided *P*≤0.004). Considering the totality of evidence from these 2 large longitudinal studies, people carrying the T2 variant (G allele for 11812) were more likely than their AMD-free peers to have advanced AMD.

We tested the independence of the T2-advanced AMD relationship by simultaneously entering A11812G with AMD susceptibility loci Y402H and A69S in age-, sex-, and smoking-adjusted logistic regression models. The distribution of A11812G did not vary by Y402H or A69S (**Table S2**). AREDS data on both Y402H and A69S existed for 601 people with advanced AMD and 132 AMD-free people. Modeling these variants had a negligible effect on our findings for T2 (OR = 5.19 (95%CI 1.84–22.69, 2-sided *P*≤0.028)). Data on Y402H existed for 52 BMES participants with advanced AMD and 238 of their AMD-free peers. The T2-advanced AMD relationship persisted after adding these data to the pooled AREDS A and AREDS B cohort (OR = 2.66 (95%CI 1.29–5.49, 2-sided *P*≤0.008). We conclude that presence of the Y402H or A69S risk allele did not alter the T2 haplogroup-advanced AMD association.

## Discussion

Our aim was to elucidate novel mtDNA-AMD relationships and *we consider our findings most valuable for exploratory purposes*. BMES was a population-based cohort and although T2 frequency was similar to AREDS in the overall sample and AMD groups, this study contributed only ∼10% of our advanced AMD cases. While members of AREDS and BMES cohorts were all of European origin we did not have specific information on ethnic background. This may partially explain our findings, as clear differences in allele frequencies exist between European populations by geographic origin of ancestry [18]. Risk estimation will be most relevant with forthcoming analyses on additional samples from population-based cohorts. Both T2-associated variants identified in our comprehensive scan are in genes encoding core subunits embedded in the hydrophobic domain of complex I (NADH ubiquinone oxidoreductase) within the oxidative phosphorylation (OXPHOS) system. Phylogenetic data and primary sequence comparisons indicate genes for these two subunits are evolutionarily conserved in mammals [19]. The A11812G variant exists at locus 351 of codon 3 of *MT-ND4*. *MT-ND4* codes for NADH dehydrogenase subunit 4 – a homologue of the *E. coli* NDH-1 proton translocation module polypeptide nuoM. NuoM shares strong sequence similarity with the antiporter multiple resistance and pH locus (Mrp) D of numerous *Bacillus* strains and for this reason is thought to have been recruited as a functional unit (instead of evolving from gene duplications). The nuoM link with this class of antiporter suggests a H^+^ channel composition and activity within proton translocation systems. *MT-ND4* has also been implicated in ubiquinone biosynthesis and is essential for complex I assembly [20]. A rare *MT-ND4* variant characterizing the T2 haplogroup existed in a small case study of people with Leber hereditary optic neuropathy (LHON), a blinding disease [21]. The A14233G variant exists at locus 147 of codon 3 of *MT-ND6* (NADH dehydrogenase subunit 6). *MT-ND6* products compose elements of the proton translocation module. Like *MT-ND4*, *MT-ND6* is essential for complex I assembly [20]. *MT*-ND6 is thought to be incorporated into complex I early in initial phases of the assembly process, while *MT*-*ND4* follows after assembly of a number of intermediate subunits [19].

Neither A11812G nor A14233G are non-synonymous substitutions. While there is currently no evidence to conclusively demonstrate that these variants may impact translation dynamics for complex I (and thereby alter the bioenergetic system of the neural and vascular retina), our results provide a reasonable basis for examining this possibility. We also note that 1–2% of the mt genome does not yield high-quality sequence data, and our resequencing algorithm is not designed to detect insertions/deletions. Thus, it is possible that the T2-associated functional variant is actually in an unsequenced region or a copy number variant, so that evaluating this possibility is a high priority. Somatic mtDNA mutations induced in the PolgA^mut^ mouse cause premature aging phenotypes without altering reactive oxygen species production, suggesting that respiratory chain dysfunction itself is the etiologic factor in many age-related diseases [22]. In addition to possible bioenergetic defects, the volume of reactive metabolites from the OXPHOS system may be altered through inherited variants and this may damage mitochondrial ultrastructure or induce redox sensitive genes implicated in cell survival and pathologic angiogenesis.

AREDS data were used in the first genome-wide association study to identify the Y402H variant of the *CFH* gene as a strong risk factor for advanced AMD [23]. We had data on Y402H in 709 cases and 373 controls for whom genotypes on T2-associated variants existed. We examined the main effects of the 402 risk allele and T2 variants in this population and then evaluated the possibility that our findings on T2 could be explained by existence of a higher frequency of the 402 risk allele among people with T2 haplotype. Main effects for Y402H with a T2 term in age-, sex-, and smoking-adjusted models (OR = 2.63, 95%CI 2.06–3.36, 2-sided *P*-value≤0.0001) were similar to those from extant reports. Results for T2 (OR = 2.66, 95%CI 1.29–5.49, 2-sided *P*-value≤0.008) were similar to those we report for data combined from AREDS A+AREDS B. In summary, modeling Y402H and T2 simultaneously did not alter the standard error of either variable and yielded a negligible change in the magnitude of ORs. A strong AMD-LOC387715 (A69S) association also exists in the AREDS cohort [24]. We had data on A69S and T2 variants for 615 cases and 136 controls and applied these for the same purposes guiding us in the Y402H analyses. The main effect of the A69S risk allele on advanced AMD (OR = 3.32, 95%CI 2.36–4.67, 2-sided *P*-value≤0.0001 in the model described above was similar to those reported in independent samples; in our analyses T2 retained significance (OR = 3.99, 95%CI 1.19–13.45, 2-sided *P*-value≤0.026). Modeling Y402H, A69S and T2 simultaneously did not impact the variance in either variable and resulted in no appreciable change to the magnitude of ORs (Y402H = 2.49, *P*≤0.001; A69S = 3.22, *P*≤0.001; T2 = 5.19 (95%CI 1.19–22.69), 2-sided *P*≤0.029). We conclude that presence of the Y402H or A69S risk allele does not alter the effect of the T2 haplogroup on advanced AMD (respective *P*-values for the Y402H*T2 and A69S*T2 interactions are ≤0.45 and ≤0.29). **Table S2** contains cross-tabulations of T2 with Y402H and A69S.

In conclusion, we used a rapid high-throughput method to determine that prevalence of two variants defining the mtDNA T2 haplogroup was higher in people with sight-threatening AMD than in their healthy peers. Both variants existed in highly conserved genes of NADH ubiquinone oxidoreductase (Complex I). If these results are substantiated by functional studies demonstrating OXPHOS deficits in people with AMD, they may provide a basis for planning clinical trials with compounds exhibiting antioxidant and mitotropic properties.

## Materials and Methods

Our inferences are based on a 3-stage research design in 406 AMD-free people and 762 people with advanced AMD. We applied a mitochondrial genome-sequence scan, evaluation of single marker- and mtDNA haplogroup-AMD associations, and validation with representative SNPs in independent cohorts examined in large prospective studies designed to systematically measure AMD endpoints. All data are from elderly self-identified white, non-Hispanic U.S. and Australian residents participating in The Age-Related Eye Disease Study (AREDS) [15] or The Blue Mountains Eye Study (BMES) [5]–[7]. The primary outcome of this report is advanced AMD, defined as presence of geographic atrophy or neovascular AMD at any time during the 12-year course of AREDS or geographic atrophy or neovascular AMD at any time during the 10-year course of BMES. We ascertained phenotype in AREDS annually from stereoscopic color fundus images using a standardized and validated protocol. For the BMES, advanced AMD was also determined from stereoscopic color fundus images using a standardized and validated protocol; data were collected at baseline and at 5- and 10-years-post enrollment for some participants. The likelihood of having advanced AMD increases 2-to-6 fold after age 74. To optimize the discovery of AMD susceptibility loci we thus selected controls from the oldest AREDS and BMES participants who were essentially free of age-related macular abnormalities. AMD-free controls had no or less than 5 small drusen (<63 um diameter) in both eyes across the 12- and 10-year follow-up periods; in addition, these people had bilateral visual acuity of 20/32 or better and were free of any clinically significant ocular pathology. Before study initiation, the AREDS protocol was approved by a Data and Safety Monitoring Committee and by the Institutional Review Board for each clinical center. Prior to enrollment, informed consent was obtained from all participants. All clinical investigation was conducted according to the principles expressed in the Declaration of Helsinki.

### Subjects and Study Design

Details of the Age-Related Eye Disease Study (AREDS) study design and methods exist elsewhere [15], [25]. AREDS was a multi-center study designed to assess the clinical course of, and risk factors for, the development and progression of AMD by collecting data on possible risk factors, measuring changes in visual acuity, photographically documenting changes in macula status, and assessing self-reported visual function. Eleven retinal specialty clinics enrolled participants aged 55 to 80 years from November 1992 through January 1998, and followed them until April 2001. A natural history study extending to December 2005 was implemented in April 2001.

Details of the Blue Mountains Eye Study (BMES) exist elsewhere [5]–[7]. BMES is a population-based survey of common eye disease and vision within a noninstitutionalized urban population aged 49 years or older. The catchment area includes 2 post-code areas of the Blue Mountains region west of Sydney, Australia. The study commenced in 1991.

### Outcomes

AREDS Report 1 contains information on the process of outcome ascertainment [25]. Advanced AMD cases met the following criteria [26]: (1) presence in either eye of geographic atrophy or neovascular AMD defined as photocoagulation or other treatment for choroidal neovascularization (based on clinical center reports); or (2) photographic documentation of any of the following; non drusenoid retinal pigment epithelial detachment, serous or hemorrhagic retinal detachment, hemorrhage under the retina or retinal pigment epithelium, and/or subretinal fibrosis either at baseline or during the course of the study. BMES cases and controls were ascertained by the methods applied in AREDS and represent analogous phenotypes. A recent BMES report provides details of outcome assessment for the advanced AMD group [5]–[7].

Our central aim was identification of AMD susceptibility loci and haplogroups. Our AMD-free group had good vision in both eyes and no existing eye disease. Controls were selected to be the oldest participants with DNA available who had no or less than 5 small drusen (<63 µm diameter) and no pigmentary abnormalities in both eyes at all visits for which fundus photographs were gradable. Both eyes had visual acuity of 20/32 or better measured by a standard protocol, ocular media was clear enough for good quality fundus photographs, and there was absence of any ocular disorder that might obscure assessment of either AMD or lens opacities.

Our controls have three distinguishing characteristics that make them the best existing comparison group for this purpose:

- Phenotype was determined annually over a 12-year period (AREDS) or across a 10-year period with a standardized protocol by multiple professional graders who were masked to phenotypic information from previous years. Adjudication with a standardized protocol occurred when discrepancies emerged.
- The criteria for AMD-free classification (<5 drusen of ≤63 µm in both eyes for the entire follow-up period) is stringent relative to those applied in previous association studies for AMD.
- The age of the AREDS AMD-free group is in the range that AMD prevalence increases ∼3 times (from ∼4% in those aged 74-to-79 to ∼12% in those aged ≥80-years) in population-based studies.

The likelihood of having AMD increases 2-to-6 fold after age 75 and it was therefore essential to select our oldest AMD-free participants to reduce the false negative rate that would otherwise result from non-random misclassification in the youngest members of the control group. The mean age (±standard error) of controls in our combined cohorts was 77.7 (±0.29) years. R. Klein and M. Knudtson at the Beaver Dam Eye Study (BDES) applied our definition of AMD-free people in their population-based sample of U.S. residents of European ancestry. Only 3.3% of the 4,628 participants were both aged 75-to-86 years and AMD-free [27]. 7.9% of BDES participants were older than 70-years-of-age and AMD-free, by our definition. It is important to note that outcome ascertainment for AREDS took place at University of Wisconsin Fundus Photograph Reading Center. Furthermore, the BMES AMD photographic grader trained at this center.

### Pooled AREDS and BMES Samples

We examined the disparity between ORs in AREDS A, AREDS B, and BMES cohorts by calculating the difference between their corresponding logarithms (log odds) and dividing this value by the square root of the standard errors of the log odds. This ratio has an approximate standard normal distribution under the hypothesis of no difference between samples, and *P*-values are computed accordingly. Results from these analyses provided an empirical basis to pool samples.

Haplotype frequency for T2 in our combined AREDS and BMES sample was 8.21% (95%CI 6.78%–9.94%); this value is within expected limits of those reported for U.S.-based cohorts of European ancestry [16]. Haplotype frequency for T2 in AMD-free participants was 5.42% (95%CI 3.61%–8.07%) reflecting the stringent nature in selection of the sample of participants in our control group. There are relatively few people without signs of AMD who are both older than 75-years-of age and of European ancestry. In The Beaver Dam Eye Study (BDES [27]), the largest population-based study on AMD in U.S. residents of European ancestry, 3.34% (95%CI 2.84%–3.94%) of the 4628 participants were both 75-to-86 years of age and AMD-free by our ascertainment criteria.

### Power Analysis

We applied the method of Samuels *et al.* [28] to derive power estimates for our combined AREDS sample. In the pooled AREDS sample there was approximately 83.5% power to detect an increase in allele frequency of the size observed assuming presence of 11 mtDNA European haplogroups and an α-level of 0.05. We report this conservative estimate, based on assumed variation in 11 European haplogroups. Power estimates at α = 0.05 for 5 haplogroups (the number defining 90% of the AREDS cohort) and 2 haplogroups (the number tested in Stage II and Stage III) yielded slight increases in power to 84.5% and 86.0%, respectively. In the pooled AREDS+BMES sample there was approximately 97.5% power to detect an increase in allele frequency of the size observed assuming presence of 11 mtDNA European haplogroups and an α-level of 0.05.

### Meta-Analysis

We used the method of DerSimonian and Laird [17] to compute combined ORs. Cochran's Q statistic for homogeneity suggested that the magnitude of effect in ORs differed only as a function of sampling error and permitted application of the fixed-effect model (Q = 3.98, df = 2, p = 0.14). Since Q is susceptible to inaccuracy due to the small number of studies analyzed we used the I^2^ index to quantify the degree of heterogeneity attributed to between-studies variation. For T2, 50% of the total variability in the magnitude of OR can be explained by actual heterogeneity between studies. On this basis we selected the point estimate derived from the random-effects model.

### Mitochondrial Resequencing

Resequencing of the entire mitochondrial genome was performed using the Affymetrix GeneChip Human Mitochondrial Resequencing Array 2.0 according to manufacturer's protocols. PCR and hybridization conditions are as previously described [29]. Grid-alignment and genotype calls were assigned using RA Tools Ver1.0.5 (http://www.dpgp.org/RA/ra.htm) based on the ABACUS algorithm under the assumption of a haploid genome [30].

### Genotyping

In the AREDS cohort the 11812G variant always existed with the 14233G variant. We believe that the likelihood that an individual would carry only one of these variants is extremely low, given the absence of this event in any of the 400 independent mitochondrial samples we have analyzed for work outside of the current study.

Genotyping for mtSNPs 4917 and 11812 were performed using TaqMan Assays by Design (Applied Biosystems). Primers and probes are listed below:

SNP Forward Primer Sequence Reverse Primer Sequence

MITO4917 GACATCCGGCCTGCTTCT TGGGTTTGGTTTAATCCACCTCA

MITO11812 ACAGTCGCATCATAATCCTCTCTCA GAGGTTAGCGAGGCTTGCT

SNP Reporter 1 Sequence Reporter 2 Sequence

MITO4917 CTCCCTCACTAAACGTAAG TCCCTCACTAGACGTAAG

MITO11812 ACTTCAAACTCTACTCCCAC TTCAAACTCTGCTCCCAC

## Supporting Information

Table S1 Demographic and life-style characteristics of AREDS and BMES samples. All participants are self-identified as being of being of non-Hispanic white origin. AMD = age-related macular degenration; Adv. AMD = advanced AMD (geographic atrophy and or neovascular) AREDS = Age-Related Eye Disease Study; BMES = Blue Mountains Eye Study; GA = geographic atrophy; NV = neovascular; stderr = standard error. AREDS A and AREDS B cohorts are independent. Demographic data are for all participants with T1 or T2 base calls.
(0.02 MB XLS)

Table S2 Cross-tabulations of percent allelic frequency in T2-associated variants for Y402H and A69S
(0.02 MB XLS)
